# Incremental Value of NT‐proBNP Over HCM‐AF Score in Risk Stratification for Atrial Fibrillation in Patients With Hypertrophic Cardiomyopathy

**DOI:** 10.1002/clc.70276

**Published:** 2026-03-20

**Authors:** Yi‐Peng Gao, Ya‐Ting Fan, Xue‐Qing Cheng, Pei‐Na Huang, Hong‐Yun Liu, Xiao‐Jun Bi, Jie Sun, Ying Zhu, Wei Zhou, Ya‐Ni Liu, You‐Bin Deng

**Affiliations:** ^1^ Department of Medical Ultrasound, Tongji Hospital, Tongji Medical College Huazhong University of Science and Technology Wuhan Huazhong China

**Keywords:** atrial fibrillation, HCM‐AF score, hypertrophic cardiomyopathy, NT‐proBNP

## Abstract

**Background:**

HCM‐AF score is a novel risk stratification tool for atrial fibrillation (AF) in hypertrophic cardiomyopathy (HCM). N‐terminal pro‐brain natriuretic peptide (NT‐proBNP) has shown promise in predicting AF. We aim to explore the incremental value of NT‐proBNP over HCM‐AF score.

**Methods:**

In this retrospective cohort study, 778 HCM patients were included. The primary endpoint was new‐onset AF. Spline curve analysis was conducted to identify the cut‐off value of NT‐proBNP. Harrell's C‐index and likelihood ratio test were conducted to explore the incremental value.

**Results:**

After a follow‐up of 3.4 ± 2.3 years, AF occurred in 65 (8.4%) patients. The cut‐off of NT‐proBNP was 240 pg/mL. Incidence rates of AF per 1000 person‐years for the low, intermediate, and high HCM‐AF score groups were 8.7 (95% confidence interval [CI]: 3.5–17.7), 18.0 (95% CI: 7.7–48.8), and 59.6 (95% CI: 27.1–157.1), respectively, with the high HCM‐AF score group significantly higher. For the low and high NT‐proBNP groups, incidence rates were 9.2 (95% CI: 4.6–16.1) and 38.1 (95% CI: 20.3–79.4), respectively. High HCM‐AF score (hazard ratio [HR]: 3.55, 95% CI: 1.33–9.48; *p* = 0.011) and high NT‐proBNP (HR: 2.49, 95% CI: 1.21–5.10; *p* = 0.013) are independent predictors for AF. Addition of NT‐proBNP improved models based on HCM‐AF score, with C‐index increasing from 0.709 to 0.768 and likelihood ratio increasing from 33.15 to 51.02.

**Conclusion:**

HCM‐AF score is reliable and robust for Asian HCM patients. NT‐proBNP demonstrated incremental value over HCM‐AF score in the prediction of new‐onset AF in patients with HCM. Future studies are warranted to incorporate HCM‐AF score and NT‐proBNP.

## Graphical abstract

In Asian patients with hypertrophic cardiomyopathy, integrating NT‐proBNP with the clinical HCM‐AF score provides significant incremental prognostic value. This combined multiparametric assessment enhances the predictive accuracy for new‐onset atrial fibrillation, enabling more precise risk stratification and targeted clinical management.

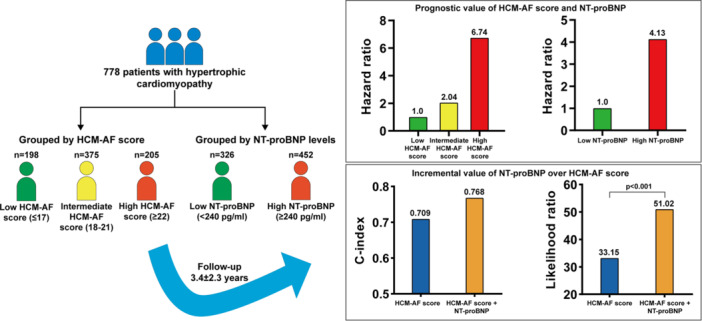

**Abbreviations**
AF: atrial fibrillation
BNP: brain natriuretic peptide
HCM: hypertrophic cardiomyopathy
LA: left atrial
LV: left ventricular
MR: mitral regurgitation
NT‐proBNP: N‐terminal pro‐B‐type natriuretic peptide
NYHA: New York Heart Association
SRT: septal reduction therapy

## 1 Introduction

Atrial fibrillation (AF) is the most common sustained arrhythmia in hypertrophic cardiomyopathy (HCM), with a reported prevalence of about 20%–25% among HCM patients and an estimated annual incidence of 2%–4% [1]. These patients have a high risk of stroke and heart failure [2, 3]. Therefore, risk stratification for AF in HCM patients is crucial for more targeted, personalized treatment and more judicious use of continuous ambulatory monitoring. Recently, a risk model specifically designed for AF prediction in HCM, called the HCM‐AF score, was developed, which consisted of left atrial (LA) diameter, New York Heart Association (NYHA) class, age at HCM diagnosis, and age at clinical evaluation [4]. However, it was observed that for the prediction of new‐onset AF, the model exhibited a sensitivity of 95% and a specificity of 25% among patients with mid‐ to high HCM‐AF scores, and a sensitivity of 58% and a specificity of 66% among those with high HCM‐AF scores [4]. Therefore, there is a need to find a method to enhance the performance of the HCM‐AF score.

Brain natriuretic peptide (BNP) and N‐terminal pro‐B‐type natriuretic peptide (NT‐proBNP), especially the latter, are important indicators in the prognosis of several cardiovascular diseases [5]. In HCM, studies have shown that BNP and NT‐proBNP levels are related to the degree of left ventricular (LV) hypertrophy, LV outflow gradient, impairments in both LV systolic and diastolic function, and LA remodeling [6, 7], all of which are predictors of AF as well. A recent study reported that both BNP and NT‐proBNP were significantly associated with the occurrence of new‐onset AF in patients with HCM [7], providing a strong rationale to investigate whether NT‐proBNP offers incremental prognostic value beyond existing clinical risk scores, such as the HCM‐AF score. Additionally, given that HCM is a heterogeneous disease, a previous study involving a population of Italian HCM patients indicated that the HCM‐AF score demonstrated relatively poor performance [8], highlighting the need for further validation of the HCM‐AF score across diverse populations. To date, the performance of the HCM‐AF score has not been specifically evaluated in an exclusively Asian population, representing an important knowledge gap. In this study, we aimed to explore the incremental value of NT‐proBNP over the HCM‐AF score for the prediction of new‐onset AF in an Asian cohort of HCM patients.

## 2 Methods

### 2.1 Study Population and Data Collection

**Figure 1:**
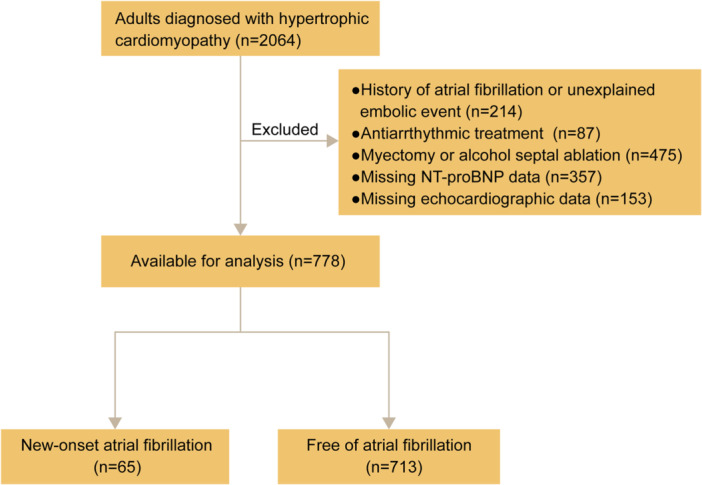
Flowchart of study population. NT‐proBNP, N‐terminal pro‐brain natriuretic peptide.

The protocol was approved by the institutional review board (TJ‐IRB202410062). Adult patients (≥ 18 years) with HCM were included, defined by a maximal LV wall thickness ≥ 15 mm in the absence of loading conditions or systemic disorders known to cause hypertrophy [9]. Patients were enrolled from January 2014 to December 2023. Patients were excluded as follows: (1) history of AF or unexplained embolic event before study entry; (2) use of antiarrhythmic medications (including Class I agents such as flecainide and propafenone, and Class III agents such as amiodarone and sotalol) to treat ventricular tachyarrhythmias; or (3) incomplete clinical data, including NT‐proBNP levels or echocardiographic records. Additionally, we excluded patients who underwent septal reduction therapy (SRT), including myectomy or alcohol ablation, during the follow‐up period, due to the significant impact of SRT on the prognosis of patients with HCM (Figure 1).

Transthoracic echocardiography was performed with GE Vivid E9 or E95 scanners (GE Healthcare, Horten, Norway) with an M5Sc transducer. LA end‐systolic diameter was obtained from the parasternal long‐axis view following guideline recommendations [10]. Maximal wall thickness was assessed at basal, mid‐ventricular, and apical levels from parasternal short‐axis views at end‐diastole. LVEF (ejection fraction) was derived using the biplane Simpson's method [10]. LV outflow tract obstruction (LVOTO) was defined as a peak systolic gradient ≥ 30 mmHg at rest or with provocation (Valsalva maneuver, exercise, or amyl nitrite) [11]. Mitral regurgitation (MR) severity was graded according to established protocols [12, 13]. Demographic and clinical variables were retrieved from electronic medical records. Height, weight, and NT‐proBNP levels measured within 1 month of the echocardiographic examination were included in the analysis.

### 2.2 HCM‐AF Score Calculation

The HCM‐AF score is calculated by summing the following components: (1) an initial score of +8 points for a LA diameter of 24–29 mm, with an additional +2 points for every 6 mm increase in LA diameter; (2) an initial score of +3 points for age of 10–19 years, with an additional +3 points for every 10‐year increase in age at a clinical evaluation; (3) a score of 0 points for age of 0–9 years, with a deduction of −2 points for every 10‐year increase in age at initial HCM diagnosis (reflecting an increased risk for patients diagnosed at younger ages); and (4) an additional +3 points for the presence of NYHA class II, III, or IV heart failure symptoms. Based on the total score, patients were categorized into three groups: low HCM‐AF score (HCM‐AF score ≤ 17), intermediate HCM‐AF score (HCM‐AF score 18‐21), and high HCM‐AF score (HCM‐AF score ≥ 22). The patient's age at this visit is used as the age at clinical evaluation, while the diagnosis age is inquired during follow‐up.

### 2.3 Follow‐up and Clinical Outcome

Follow‐up procedures were conducted according to our previously reported institutional protocol [14]. Briefly, follow‐up was initiated at the time of the first documented visit confirming the diagnosis of HCM in patients with complete clinical records. Outcome data were obtained from electronic medical records or telephone contact to the patients or their relatives. Patients were followed until the occurrence of new‐onset AF, all‐cause death, or the study's end in November 2024.

The primary endpoint was new‐onset AF, defined as ≥ 1 clinically overt episode without distinct P‐waves documented by electrocardiogram, Holter electrocardiogram monitoring, telemetry, or after expert analysis of device recordings in patients with implantable cardiac monitoring systems [15]. Brief asymptomatic AF episodes that were fortuitously identified through ambulatory electrocardiogram were excluded from the analysis [16].

### 2.4 Statistical Analyses

Continuous variables were summarized as mean ± standard deviation or median (interquartile range), depending on distribution, while categorical variables were presented as counts and percentages. Between‐group comparisons were performed using Student's *t*‐test, Mann−Whitney *U* test, Chi‐square test, or analysis of variance as appropriate. NT‐proBNP levels were converted to log10 and expressed as LgNT‐proBNP.

The NT‐proBNP cut‐off value recommended by the guidelines is used for the diagnosis of heart failure [17]. To investigate the relationship between NT‐proBNP levels and the risk of new‐onset AF, a spline curve analysis [18] was conducted to investigate the hazard ratio (HR) changes for new‐onset AF across the range of LgNT‐proBNP values. The spline model used three knots placed at the 10th, 50th, and 90th percentiles of LgNT‐proBNP to capture nonlinear associations. The value of LgNT‐proBNP corresponding to an HR of 1, taken from the lower limit of the 95% confidence intervals (CI), is established as the cut‐off value, the threshold above which NT‐proBNP was associated with a higher risk of new‐onset AF in this HCM cohort. AF‐free survival was analyzed using Kaplan–Meier curves and compared across groups using the log‐rank test. Patients lost to follow‐up were treated as censored observations. Cox proportional hazards models were applied to identify predictors of new‐onset AF. Variables associated with outcomes in univariable analyses were entered into multivariable models. To evaluate the incremental prognostic value of NT‐proBNP over the HCM‐AF score, Harrell's C‐index and likelihood ratio tests were conducted. Furthermore, a subgroup analysis was performed in patients without severe MR to assess the robustness of the findings.

All analyses were performed using R software (version 4.3.3, R Project for Statistical Computing, Vienna, Austria). A two‐sided *p* < 0.05 was considered statistically significant.

## 3 Results

### 3.1 Baseline Characteristics

Initially, a total of 2064 patients were enrolled in our hospital's medical record server. After exclusions, 778 patients were included for analysis, of whom 65 experienced new‐onset AF (Figure 1). Baseline clinical characteristics of the overall population, those with or without new‐onset AF are reported in Table 1. Compared to the patients without AF, patients with new‐onset AF were older at clinical evaluation (61.0 ± 10.2 vs. 56.1 ± 12.8, *p* = 0.003), had a higher proportion of females (32 [49.2] vs. 230 [32.3], *p* = 0.008), more frequent severe MR (12 [18.5] vs. 36 [5.0], *p* < 0.001), and NYHA class > 1 (37 [56.9] vs. 166 [23.3], *p*< 0.001), while the proportion of hypertension was significantly lower (31 [47.7] vs. 448 [62.8], *p* = 0.023) (Table 1). Significantly larger LA diameter (42.3 ± 7.3 vs. 39.0 ± 6.1, *p* < 0.001), higher E/e' ratio (18.0 ± 9.2 vs. 15.6 ± 6.6, *p* = 0.007), and higher NT‐proBNP levels (1113 [389–2173] vs. 305 [105–974], *p* < 0.001) were observed in patients with new‐onset AF as well (Table 1). Age at HCM diagnosis, body mass index, LVEF, maximum LV wall thickness, and the proportions of coronary artery disease, diabetes, and LVOTO were comparable between these two groups (Table 1).

**Table 1:** Baseline characteristics of study population.

	Overall	No AF	New‐onset AF	*p* value
	*n* = 778	*n* = 713	*n* = 65	
**Age at HCM diagnosis, years**	49.7 ± 13.0	49.4 ± 13.1	52.4 ± 12.5	0.078
**Age at clinical evaluation, years**	56.5 ± 12.7	56.1 ± 12.8	61.0 ± 10.2	0.003
Female	262 (33.7)	230 (32.3)	32 (49.2)	0.008
Body mass index, kg/m^2^	25.1 ± 3.6	25.2 ± 3.6	24.8 ± 2.9	0.471
Coronary artery disease	348 (44.7)	319 (44.7)	29 (44.6)	1.0
Hypertension	479 (61.6)	448 (62.8)	31 (47.7)	0.023
Diabetes	175 (22.5)	157 (22.0)	18 (27.7)	0.372
LVOTO	247 (31.7)	228 (32.0)	19 (29.2)	0.752
Severe MR	48 (6.2)	36 (5.0)	12 (18.5)	< 0.001
**NYHA class > 1**	203 (26.1)	166 (23.3)	37 (56.9)	< 0.001
LVEF, %	64.9 ± 6.2	65 ± 6.2	63.3 ± 6.5	0.089
Maximum LV wall thickness, mm	17.5 ± 3.8	17.5 ± 3.8	17.7 ± 3.1	0.686
**LA diameter, mm**	39.3 ± 6.3	39.0 ± 6.1	42.3 ± 7.3	< 0.001
**E/e' ratio**	15.8 ± 6.9	15.6 ± 6.6	18.0 ± 9.2	0.007
HCM‐AF score	19.7 ± 3.3	19.4 ± 3.2	22.4 ± 3.5	< 0.001
NT‐proBNP, pg/mL	335 (114–1091)	305 (105–974)	1113 (389–2173)	< 0.001
LgNT‐proBNP	2.56 ± 0.75	2.52 ± 0.75	3.01 ± 0.56	< 0.001

*Note:* Values are mean ± SD, *n* (%), or median (Q1–Q3). Components of the HCM‐AF score were bolded.

Abbreviations: AF, atrial fibrillation; HCM, hypertrophic cardiomyopathy; LA, left atrial; LVOTO, ventricular outflow tract obstruction; MR, mitral regurgitation; NT‐proBNP, N‐terminal pro‐brain natriuretic peptide; NYHA, New York Heart Association.

### 3.2 Survival Outcomes

**Figure 2:**
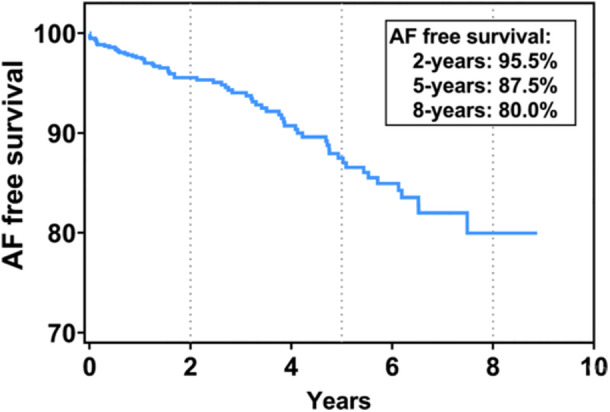
Kaplan−Meier curve for atrial fibrillation‐free survival in the study population. AF, atrial fibrillation.

**Figure 3:**
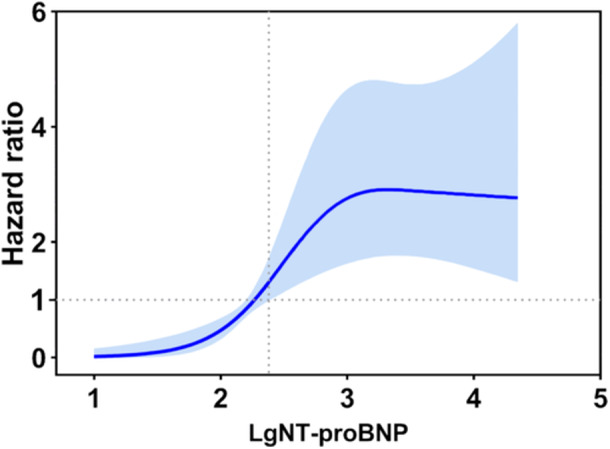
Spline curve analysis for new‐onset atrial fibrillation according to LgNT‐proBNP values. Restricted cubic spline illustrates the changes in hazard ratios for new‐onset atrial fibrillation across the range of LgNT‐proBNP values, with the shaded blue areas representing the 95% confidence intervals. The intersection of the lower limit of the 95% confidence intervals with the hazard ratio of 1 occurs at an LgNT‐proBNP value of 2.38. NT‐proBNP, N‐terminal pro‐brain natriuretic peptide.

During a mean follow‐up of 3.4 ± 2.3 years, 65 (8.4%) patients experienced new‐onset AF. The survival rates free from AF at 2, 5, and 8 years were 95.5%, 87.5%, and 80.0%, respectively (Figure 2), with an annual rate of 2.5%. Following the calculation of HCM‐AF score, the numbers of patients in the low (≤ 17), intermediate (18–21), and high (≥ 22) HCM‐AF score groups were 198 (25.5%), 375 (48.2%), and 205 (26.3%), respectively. The spline curve illustrating the relationship between LgNT‐proBNP and the occurrence of new‐onset AF is presented in Figure 3. A cut‐off value for LgNT‐proBNP was 2.38, which corresponds to an NT‐proBNP level of approximately 240 pg/mL (Figure 3). High NT‐proBNP level were observed in 452 (58.1%) patients.

**Figure 4:**
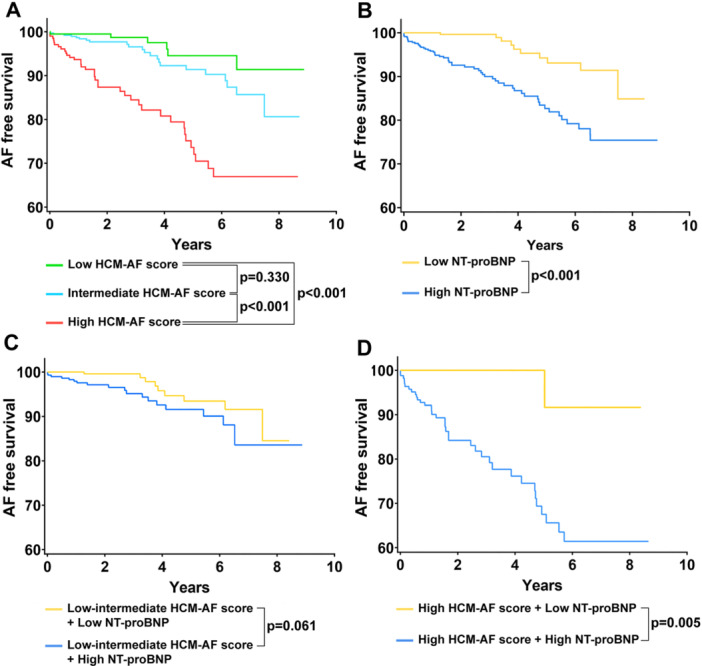
Kaplan−Meier curves for new‐onset atrial fibrillation stratified by HCM‐AF score and NT‐proBNP cut‐off value. (A) Patients stratified by HCM‐AF score: low (≤ 17), intermediate (18–21), and high (≥ 22). (B) Patients stratified by NT‐proBNP cut‐off value. (C) Patients with low‐intermediate HCM‐AF score further stratified by NT‐proBNP cut‐off value. (D) Patients with high HCM‐AF score further stratified by NT‐proBNP cut‐off value. The cut‐off value for NT‐proBNP is 240 pg/mL. NT‐proBNP, N‐terminal pro‐brain natriuretic peptide.

The outcomes of the patients are summarized in Table 2. New‐onset AF occurred in 6 (3.0%), 22 (5.9%), and 37 (18.0%) patients in low, intermediate, and high HCM‐AF score groups, respectively. A significantly higher new‐onset AF event rate per 1000 person‐years was observed in the high HCM‐AF score group, while the rate was comparable between the low and intermediate HCM‐AF score groups (Table 2). Regarding the NT‐proBNP, new‐onset AF was observed in 10 (3.1%) and 55 (12.2%) patients in the low and high NT‐proBNP groups, respectively, and the new‐onset AF event rate per 1000 person‐years was significantly higher in the high NT‐proBNP group (Table 2). Cumulative event‐free survival analyses for new‐onset AF are illustrated in Figure 4. Patients with high HCM‐AF scores exhibited significantly worse event‐free survival rates (Figure 4A). A similar trend was noted in the low and high NT‐proBNP groups, where patients with high NT‐proBNP level also showed significantly worse event‐free survival (Figure 4B).

**Table 2:** Outcome of the study population stratified by HCM‐AF score or NT‐proBNP.

	HCM‐AF score			NT‐proBNP	
	Low	Intermediate	High	Low	High
	*n* = 198	*n* = 375	*n* = 205	*n* = 326	*n* = 452
New‐onset AF events	6 (3.0)	22 (5.9)	37 (18.0)	10 (3.1)	55 (12.2)
Incidence (95% CI) per 1000 person‐years	8.7 (3.5–17.7)	18.0 (7.7–48.8)	59.6 (27.1–157.1)*,#	9.2 (4.6–16.1)	38.1 (20.3–79.4)*

*Note:* Values are median (Q1–Q3) or event rate (95% CI). The cut‐off value for NT‐proBNP is 240 pg/mL. *p < 0.05 versus low HCM‐AF score or low NT‐proBNP; ^#^ *p* < 0.05 versus intermediate HCM‐AF score.

Abbreviations: CI, confidence interval; other abbreviations as in Table 1.

Univariable and multivariable Cox regression analyses for new‐onset AF are summarized in Table 3. The univariable Cox regression analysis indicated that female sex, severe MR, E/e' ratio, LA diameter, high HCM‐AF score, and high NT‐proBNP level were associated with new‐onset AF. Following multivariable Cox regression analysis, severe MR (HR 2.52, 95% CI 1.31–4.85; *p* = 0.006), high HCM‐AF score (HR 3.55, 95% CI 1.33–9.48; *p* = 0.011), and high NT‐proBNP level (HR 2.49, 95% CI 1.21–5.10; *p* = 0.013) remained independently associated with new‐onset AF (Table 3).

**Table 3:** Univariate and multivariate Cox regression for new‐onset atrial fibrillation.

	Univariate		Multivariate	
	Hazard ratio (95% CI)	*p* value	Hazard ratio (95% CI)	*p* value
Female	2.0 (1.23–3.25)	0.005	1.54 (0.93–2.56)	0.094
Hypertension	0.69 (0.42–1.12)	0.135		
Severe MR	4.54 (2.41–8.55)	< 0.001	2.52 (1.31–4.85)	0.006
E/e' ratio	1.05 (1.02–1.08)	< 0.001	1.01 (0.97–1.04)	0.624
LA diameter	1.09 (1.05–1.12)	< 0.001	1.03 (0.99–1.08)	0.153
HCM‐AF score	1.30 (1.21–1.39)	< 0.001		
18–21	2.04 (0.83–5.04)	0.121	1.85 (0.73–4.64)	0.193
> 21	6.74 (2.84–15.98)	< 0.001	3.55 (1.33–9.48)	0.011
LgNT‐proBNP	2.13 (1.57–2.88)	< 0.001		
> 2.38	4.13 (2.11–8.10)	< 0.001	2.49 (1.21–5.10)	0.013

Abbreviations: CI, confidence interval; other abbreviations as in Table 1.

In the subgroup of patients without severe MR, Kaplan−Meier curves showed significantly worse event‐free survival for patients with a high HCM‐AF score and for those with a high NT‐proBNP level (Supporting Information S1: Figure S1A and S1B). The univariable Cox regression analysis indicated that female sex, severe MR, E/e' ratio, LA diameter, high HCM‐AF score, and high NT‐proBNP level were associated with new‐onset AF (Supporting Information S1: Table S1). High HCM‐AF score (HR 3.12, 95% CI 1.14–8.52; *p* = 0.027) and high NT‐proBNP level (HR 2.86, 95% CI 1.35–6.07; *p* = 0.006) remained independently associated with new‐onset AF in the multivariable Cox regression analysis.

### 3.3 Incremental Value of NT‐proBNP Over HCM‐AF Score in AF Risk Stratification

Given the similar incidence of new‐onset AF in the low and intermediate HCM‐AF score groups (Figure 4A, Table 2), these two groups were combined into a single low‐intermediate HCM‐AF score group for further analysis. Within this low‐intermediate group, patients with low NT‐proBNP level showed slightly improved event‐free survival compared to those with high NT‐proBNP level, although this difference did not reach statistical significance (*p* = 0.061, Figure 4C). Patients with high HCM‐AF score plus low NT‐proBNP level showed significantly improved event‐free survival compared to patients with high HCM‐AF score plus high NT‐proBNP level (*p* = 0.005, Figure 4D).

**Figure 5:**
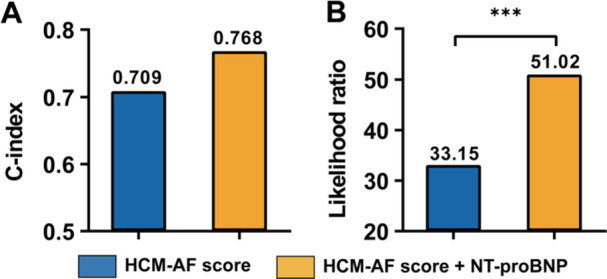
Prognostic and incremental value of NT‐proBNP with Harrell's C‐index and likelihood ratio test. (A) Harrell's C‐index for the HCM‐AF score and the HCM‐AF score plus NT‐proBNP. (B) Likelihood ratio test for the HCM‐AF score and the HCM‐AF score plus NT‐proBNP. NT‐proBNP, N‐terminal pro‐brain natriuretic peptide. ***p < 0.001.

Cox regression models, based on HCM‐AF score (Model 1) and the combination of HCM‐AF score and NT‐proBNP classification (Model 2), were employed to further assess the incremental value of NT‐proBNP. When NT‐proBNP classification was incorporated, the C‐index of the model based solely on the HCM‐AF score increased from 0.709 to 0.768, and the likelihood ratio rose from 33.15 to 51.02 (Figure 5). In low‐intermediate HCM‐AF score and high HCM‐AF score groups, Model 2 showed an improved performance compared to Model 1 as well (Table 4).

**Table 4:** Subgroup analysis of model performance for predicting new‐onset atrial fibrillation.

	C‐Index	Likelihood
Patients without severe mitral regurgitation (*n* = 730)		
Model 1	0.701	25.61
Model 2	0.768	40.38
*p* value		< 0.001
Low‐intermediate HCM‐AF score classification (*n* = 573)		
Model 1	0.522	0.73
Model 2	0.675	5.18
*p* value		0.035
High HCM‐AF score classification (*n* = 205)		
Model 1	0.567	4.84
Model 2	0.669	18.96
*p* value		< 0.001

*Note:* Model 1, Cox proportional hazards model including the HCM‐AF score as the sole predictor. Model 2, Cox proportional hazards model including both the HCM‐AF score and NT‐proBNP classification. *p* value is derived from the Likelihood ratio test, comparing the overall fit of Model 2 against Model 1. The cut‐off value for NT‐proBNP is 240 pg/mL.

Similar trends were observed in patients without severe MR. In this subgroup, patients with low‐intermediate HCM‐AF score plus low NT‐proBNP level showed slightly improved event‐free survival compared to patients with low‐intermediate HCM‐AF score plus high NT‐proBNP level, with the difference approaching statistical significance (*p *= 0.052, Supporting Information S1: Figure S1C). Among patients with a high HCM‐AF score, those with a low NT‐proBNP level showed significantly improved event‐free survival compared to their counterparts with a high NT‐proBNP level (*p *= 0.012, Supporting Information S1: Figure S1D). For this subgroup, compared to Model 1, the C‐index increased from 0.701 to 0.768 in Model 2, and the likelihood ratio rose from 25.61 to 40.38 (Table 4).

## 4 Discussion

In the present study, we evaluated the incremental prognostic value of NT‐proBNP for new‐onset AF in patients with HCM over HCM‐AF score in Asian HCM patients. Furthermore, to the best of our knowledge, this study is the first validation of the HCM‐AF score in this specific population. The main findings of this study are that, in HCM patients: (1) HCM‐AF score demonstrates significant prognostic value for new‐onset AF in Asian patients; (2) NT‐proBNP provides additional value beyond the HCM‐AF score, thereby improving risk stratification for new‐onset AF.

### 4.1 Assessment of AF in HCM

AF is the most common sustained tachyarrhythmia in HCM, occurring in 20%–25% of HCM patients, with an incidence rate of 2%–4% per year [19, 20]. In the present study, we observed an incidence of 20.0% over 8 years of follow‐up, with a yearly incidence of 2.5% (Figure 2). AF is considered a severe and progressive feature of HCM, associated with significant mortality and morbidity [1]. HCM patients with AF face a higher risk of embolic strokes and refractory heart failure, exhibiting a two‐ to threefold increase in cardiovascular mortality rates [21]. Timely treatment, including catheter ablation and antiarrhythmic medications, has proven effective in improving patient outcomes and enhancing quality of life [9]. Therefore, it is crucial to identify patients at high risk for AF and to implement more frequent electrocardiographic screenings to ensure prompt intervention.

Predictors of clinically significant AF include LA enlargement, older age, longer HCM duration, and NYHA functional class III−IV heart failure [9]. However, the predictive performance of models based on LA diameter has shown notable variability across studies, influenced heavily by the analytical methodology. Studies using a single, dichotomous cutoff (LA diameter ≥ 45 mm) indicated relatively modest performance, with C‐indices of 0.58 [4] and 0.63 [22] respectively. In contrast, a recent Italian study demonstrated that when LA diameter was assessed as a continuous variable, it yielded a higher C‐index of 0.70 [8]. This discrepancy not only highlights the limitations of a single‐parameter approach but also reveals how its predictive power can be diluted by relying on a rigid cutoff value. This strongly underscores the need for a more comprehensive, multiparametric evaluation to achieve robust and generalizable risk stratification. Furthermore, reflecting the unique pathophysiological complexity of HCM, models developed for the general cardiovascular population, such as C_2_HEST and CHARGE‐AF, also exhibited inadequate performance, with C‐indices ranging from 0.58 to 0.64 [4]. In recent years, the HCM‐AF score was developed based on a cohort of 1900 patients with HCM and was externally validated, achieving improved performance with a C‐index of 0.70 [4]. In the present study, we assessed the performance of the HCM‐AF score in 778 Asian HCM patients, and similar results were observed, yielding a C‐index of 0.709 (Figure 5). Although a previous study involving an Italian HCM population reported a C‐index of 0.673 for the HCM‐AF score [8], overall, the HCM‐AF score proves to be a practical and robust tool for predicting the risk of new‐onset AF in patients with HCM.

Notably, we observed that patients with low and intermediate HCM‐AF scores showed no differences in the occurrence of new‐onset AF in the present study (Table 2). This may be ascribed to demographic and racial differences between our study population and cohort in which HCM‐AF score was originally developed [4]. The participants of our study are completely composed of Asian patients, which represents 4.1% of the participants in the study of Carrick et al. [4]. The established cutoffs, derived from a predominantly non‐Asian population, may therefore be less sensitive for stratifying risk at the lower end of the spectrum in Asian patients. This suggests that the cutoff values of the HCM‐AF score may need to be adjusted for Asian HCM patients to stratify low and intermediate‐risk patients.

However, while these findings highlight the potential need for population‐specific calibration of the HCM‐AF score in Asian patients, the external applicability of our results within Asia should be interpreted in the context of our study design. Our cohort was derived from a single tertiary referral center in China, and clinical characteristics, referral patterns, and management strategies may vary across regions. Therefore, our findings are most directly applicable to similar tertiary‐care East Asian settings. Future multicenter studies across diverse Asian populations will be important to determine whether region‐specific recalibration of the HCM‐AF score is warranted.

### 4.2 Association of AF and BNP

BNP is a product of neurohormonal activation that is produced and secreted almost exclusively by ventricular myocardial cells, likely in response to increases in end‐diastolic pressure and volume [23]. NT‐proBNP, on the other hand, is the N‐terminal fragment of its prohormone [24]. In patients with HCM, BNP and NT‐proBNP levels have been found to correlate with the degree of LV hypertrophy, LV outflow gradient, as well as impairments in both LV systolic and diastolic function, and LA remodeling [6, 7]. The prognostic value of natriuretic peptides for predicting new‐onset AF in patients with HCM has been clarified. A recent study of 701 HCM patients found that BNP and NT‐proBNP were significantly associated with the incidence of AF (HR of 1.30 and 1.38 for BNP and NT‐proBNP, respectively; all *p* < 0.05) [7]. Consistent with these findings, in the present study, NT‐proBNP demonstrated prognostic value for new‐onset AF, with a statistically significant HR of 2.49 for high NT‐proBNP classification (Table 3). It is reasonable to consider NT‐proBNP as an independent predictor for new‐onset AF in HCM, as elevated NT‐proBNP levels reflect adverse remodeling of LA and impairments in cardiac function, both of which have been validated as contributors to AF [7].

In the present study, we used a restricted cubic spline analysis to define a clinically relevant threshold for NT‐proBNP in our cohort. Our analysis identified that the risk for AF begins to increase significantly above an LgNT‐proBNP value of 2.38 (corresponding to 240 pg/mL). The clinical justification for this specific threshold is primarily rooted in the underlying pathophysiology of AF development in HCM, especially given that current clinical guidelines do not offer specific NT‐proBNP thresholds for predicting incident AF [9], and comparable studies in HCM are scarce. Patients with HCM inherently exhibit myocardial hypertrophy and diastolic dysfunction, leading to chronically elevated ventricular wall stress and thus higher baseline NT‐proBNP levels compared to the general population [25].

Therefore, a simple diagnostic cutoff (such as those for heart failure) is less relevant here. Instead, our data‐driven threshold of 240 pg/mL likely represents a second hit. It identifies the level above the already elevated HCM baseline at which the LA has likely undergone sufficient pathological remodeling to become a vulnerable substrate for initiating AF. Nevertheless, this cut‐off is exploratory and requires prospective validation in other cohorts before it can be broadly adopted in clinical practice.

It is worth noting that, in the overall cohort, we observed that the C‐index increased from 0.709 to 0.768 and the likelihood ratio rose from 33.15 to 51.02, suggesting that NT‐proBNP had incremental value for predicting new‐onset AF (Figure 5). Additionally, the addition of NT‐proBNP classification significantly improved the performance for patients in both the low‐intermediate HCM‐AF score (≤ 21) and high HCM‐AF score (≥ 22) groups (Table 4). This indicates that incorporating the NT‐proBNP classification can identify a subset of potentially high new‐onset AF risk patients within the low‐intermediate HCM‐AF score group, while also identifying individuals with a relatively lower new‐onset AF risk within the high HCM‐AF score group. Severe MR is a major confounder, as it can independently drive LA enlargement and elevate NT‐proBNP levels. In the subgroup of patients without severe MR, both the HCM‐AF score and the NT‐proBNP classification demonstrated robust performance (Supporting Information S1: Table S1 and Figure S1), and a similar incremental value was observed when NT‐proBNP classification was added (Table 4), underscoring the robustness of these findings.

Nowadays, both the HCM‐AF score and NT‐proBNP levels can be easily and repeatedly assessed in routine clinical practice. Our findings encourage the integration of the HCM‐AF score and NT‐proBNP level to facilitate a more precise risk stratification for new‐onset AF in patients with HCM. This approach can guide targeted clinical interventions, such as intensified electrocardiogram monitoring (e.g., prolonged Holter monitoring or wearable devices) for early AF detection in intermediate‐ or high‐risk patients, or consideration of early anticoagulation to mitigate stroke risk in those identified as high‐risk. The accessibility and affordability of NT‐proBNP testing further enhance the applicability of this model, making it a practical tool for routine use in diverse clinical settings to optimize patient management and improve outcomes.

### 4.3 Limitations

There are several limitations to our study. First, all baseline data were obtained retrospectively, which introduces inherent selection bias and therefore requires prospective validation. Second, a potential for additional selection bias exists, as our study included only patients with available NT‐proBNP measurements and complete echocardiographic data, who may not be fully representative of the broader HCM population. Third, the follow‐up period of our study is relatively short, with new‐onset AF occurring in only 65 (8.4%) cases, which may limit statistical power and increase the uncertainty of effect estimates in multivariable modeling. Larger cohorts with more outcome events are needed to confirm the robustness of these associations. Fourth, our study was conducted in an exclusively Asian cohort, which may limit the generalizability of our findings to non‐Asian populations. Furthermore, the identified NT‐proBNP cut‐off of 240 pg/mL was derived from our specific study population using restricted cubic spline analysis. Since NT‐proBNP levels are significantly influenced by age, renal function, and LV systolic function, this threshold may not be universally applicable and necessitates further external validation. Moreover, we excluded patients who underwent SRT, and the prognostic assessment for SRT patients is crucial as well. Future research is warranted to explore the prognostic value of NT‐proBNP and the HCM‐AF score for patients with HCM following SRT.

## 5 Conclusion

In conclusion, the HCM‐AF score is reliable and robust for Asian HCM patients. Integrating the HCM‐AF score with NT‐proBNP significantly enhances the risk stratification for new‐onset AF. Future studies are warranted to incorporate NT‐proBNP into HCM‐AF score to develop a new algorithm for risk stratification for AF in patients with HCM.

## Author Contributions

**Yi‐Peng Gao:** writing – original draft, formal analysis, data curation. **Ya‐Ting Fan:** writing – review and editing, data curation. **Xue‐Qing Cheng:** writing – review and editing. **Pei‐Na Huang:** writing – review and editing. **Hong‐Yun Liu:** writing – review and editing. **Xiao‐Jun Bi:** data curation. **Jie Sun:** data curation. **Ying Zhu:** data curation. **Wei Zhou:** data curation. **Ya‐Ni Liu:** data curation, resources. **You‐Bin Deng:** conceptualization, writing – review and editing, project administration, formal analysis.

## Funding

The authors received no specific funding for this work.

## Ethics Statement

This study was approved by the Ethics Committee of Tongji Hospital, Tongji Medical College, Huazhong University of Science and Technology (TJ‐IRB202410062).

## Consent

The authors have nothing to report.

## Conflicts of Interest

The authors declare no conflicts of interest.

## Supporting information


**Figure S1:** Kaplan‐Meier curves for new‐onset atrial fibrillation stratified by HCM‐AF score and NT‐proBNP cut‐off value in patients without severe mitral regurgitation. **Table S1:** Univariate and Multivariate Cox Regression for New‐Onset Atrial Fibrillation in Patients without Severe Mitral Regurgitation.

## Data Availability

The data are available from the corresponding author upon reasonable request.
