# Supplementary material for: Incremental Value of NT‐proBNP Over HCM‐AF Score in Risk Stratification for Atrial Fibrillation in Patients With Hypertrophic Cardiomyopathy
Source: Clin Cardiol. 2026 Mar 20;49(3):e70276. doi: 10.1002/clc.70276 (PMC13093646; doi:10.1002/clc.70276)
Supplement: Supplementary file 1 — Figure S1: Kaplan‐Meier curves for new‐onset atrial fibrillation stratified by HCM‐AF score and NT‐proBNP cut‐off value in patients without severe mitral regurgitation. Table S1: Univariate and Multivariate Cox Regression for New‐Onset Atrial Fibrillation in Patients without Severe Mitral Regurgitation. [file CLC-49-e70276-s001.docx]

Table S1. Univariate and Multivariate Cox Regression for New-Onset Atrial Fibrillation in Patients without Severe Mitral Regurgitation.

|  | Univariate | |  | Multivariate | |
| --- | --- | --- | --- | --- | --- |
|  | Hazard ratio (95% CI) | P-value |  | Hazard ratio (95% CI) | P-value |
| Female | 1.75(1.02-3.01) | 0.042 |  | 1.45(0.83-2.54) | 0.192 |
| Hypertension | 0.71(0.41-1.22) | 0.211 |  |  |  |
| E/e’ ratio | 1.05(1.02-1.09) | 0.002 |  | 1.01(0.97-1.05) | 0.665 |
| LA diameter | 1.08(1.04-1.12) | <0.001 |  | 1.03(0.98-1.08) | 0.196 |
| HCM-AF score | 1.22(1.13-1.31) | <0.001 |  |  |  |
| 18-21 | 1.79(0.71-4.48) | 0.214 |  | 1.69(0.66-4.31) | 0.273 |
| >21 | 5.38(2.23-13.0) | <0.001 |  | 3.12(1.14-8.52) | 0.027 |
| LgNT-proBNP | 2.05(1.47-2.86) | <0.001 |  |  |  |
| >2.38 | 3.97(1.94-8.12) | <0.001 |  | 2.86(1.35-6.07) | 0.006 |

CI, confidence interval; LA, left atrial; NT-proBNP, N-terminal pro-brain natriuretic peptide.


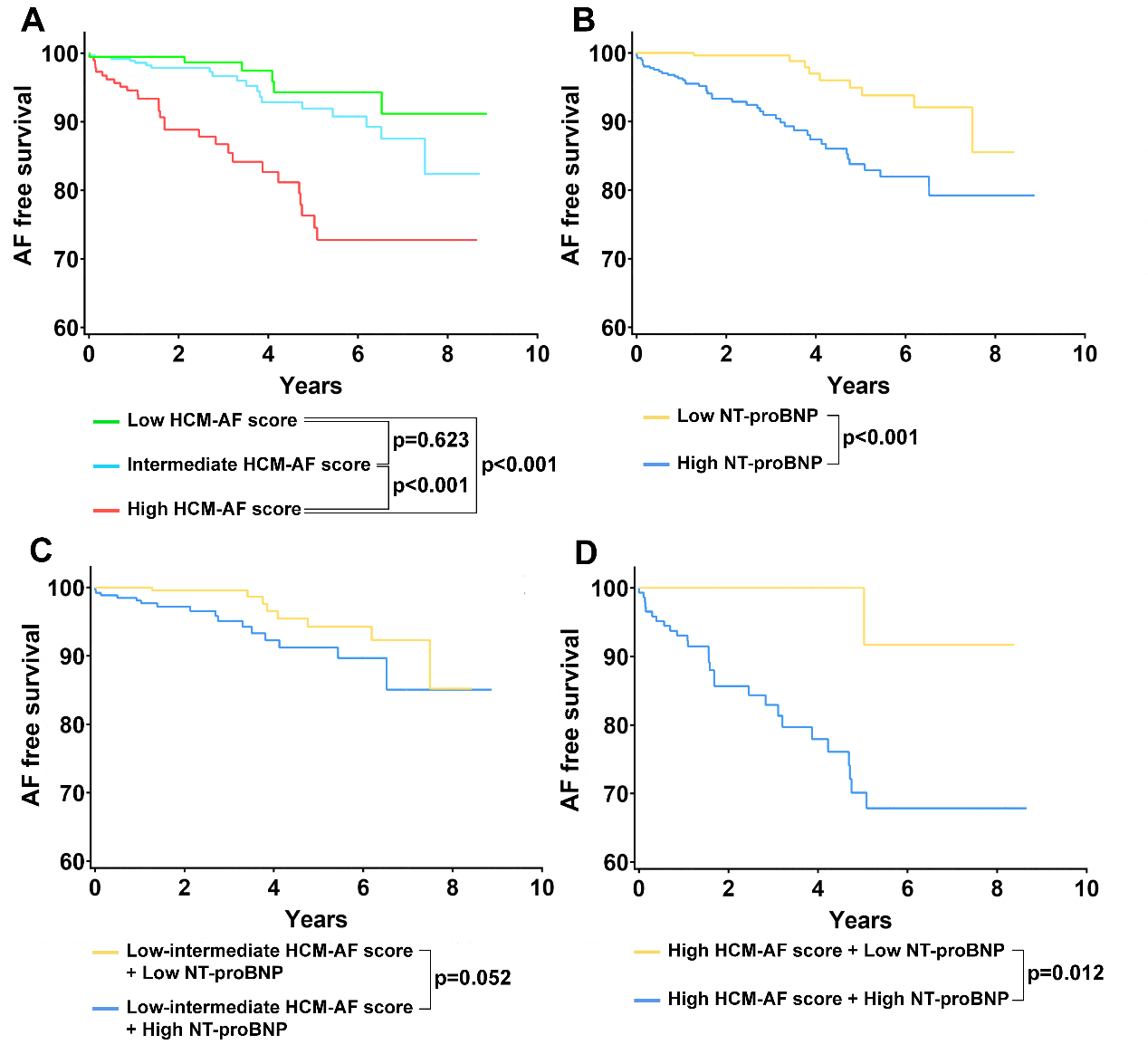


**Figure S1.** Kaplan-Meier curves for new-onset atrial fibrillation stratified by HCM-AF score and NT-proBNP cut-off value in patients without severe mitral regurgitation. (A) Patients stratified by HCM-AF score: low (≤17), intermediate (18-21), and high (≥22). (B) Patients stratified by NT-proBNP cut-off value. (C) Patients with low-intermediate HCM-AF score further stratified by NT-proBNP cut-off value. (D) Patients with high HCM-AF score further stratified by NT-proBNP cut-off value. NT-proBNP, N-terminal pro-brain natriuretic peptide. The cut-off value for NT-proBNP is 240 pg/ml.
